# The matching quality of experimental and control interventions in blinded pharmacological randomised clinical trials: a methodological systematic review

**DOI:** 10.1186/s12874-016-0111-9

**Published:** 2016-02-13

**Authors:** Segun Bello, Maoling Wei, Jørgen Hilden, Asbjørn Hróbjartsson

**Affiliations:** 1 The Nordic Cochrane Centre, Rigshospitalet, Blegdamsvej 9, 2100 Copenhagen Ø, Denmark; 2 Department of Epidemiology and Medical Statistics, College of Medicine, University of Ibadan/Ibadan Centre for Evidence-based Medicine, University College Hospital, Ibadan, Nigeria; 3 The Chinese Cochrane Centre, West China Hospital, Sichuan University, No. 37 Guo Xue Xiang, Chengdu, Sichuan 610041 P.R. China; 4 Department of Biostatistics, University of Copenhagen, Copenhagen, Denmark; 5 Centre for Evidence-based Medicine, University of Southern Denmark/Odense University Hospital, Odense, Denmark

## Abstract

**Background:**

Blinding is a pivotal method to avoid bias in randomised clinical trials. In blinded drug trials, experimental and control interventions are often designed to be matched, i.e. to appear indistinguishable. It is unknown how often matching procedures are inadequate, so we decided to systematically identify and analyse studies of matching quality in drug trials. Our primary objective was to assess the proportion of studies that concluded that the matching was inadequate; our secondary objective was to describe mechanisms for inadequate matching.

**Methods:**

Systematic review. We searched PubMed, Google Scholar and Web of Science Citation Index for studies that assessed whether supposedly indistinguishable interventions (experimental and control) in randomized clinical drug trials could be distinguished based on physical properties (e.g. appearance or smell). Two persons decided on study eligibility and extracted data independently. Our primary analysis was based on the conclusions of each study. In supportive analyses, we defined a low and a high threshold for inadequate matching. We summarised results qualitatively.

**Results:**

We included studies of 36 trials, of which 28 (78 %) were published before 1977. The studies differed considerably with regard to design, methodology and analysis. Sixteen of the 36 studies (44 %) concluded inadequate matching. When we adapted high or low thresholds for inadequate matching, the number of trials with inadequate matching was reduced to 12 (33 %) or increased to 26 (72 %). Inadequate matching was concluded in 7 of 22 trials (32 %) based on a defined cohort of trials. Inadequate matching was concluded in 9 of 14 trials (64 %) which were not based on a trial cohort, and therefore at a higher risk of publication bias. The proportion of inadequate matching did not seem to depend on publication year. Typical mechanisms of inadequate matching were differences in taste or colour.

**Conclusion:**

We identified matching quality studies of 36 randomized clinical drug trials. Sixteen of the 36 studies (44 %) concluded inadequate matching. Few studies of matching quality in contemporary trials have been published, but show similar results as found for older trials. Inadequate matching in drug trials may be more prevalent than commonly believed.

**Electronic supplementary material:**

The online version of this article (doi:10.1186/s12874-016-0111-9) contains supplementary material, which is available to authorized users.

## Background

Blinding is a pivotal methodological principle in randomised clinical trials [1, 2]. Blinding reduces the risk of bias in a trial by masking which intervention is experimental and which is control. The degree of bias in trials with nonblinded patients, treatment providers or outcome assessors can be pronounced [3–7]. For example, lack of blinding of outcome assessors exaggerates odds ratios by approximately 36 %, on average in trials with subjective outcomes (i.e. involving assessor judgment) [4].

Pharmacological trials constitute approximately 75 % of conducted trials [8], and have a profound influence on clinical medicine. A blinded drug trial typically compares an experimental drug intervention with a matched control intervention, i.e. one that appears identical to the experimental intervention, but which does not contain the essential component of the drug under investigation. Matching is implemented in more than 90 % of pharmacological trials which report blinding methods [9]. Matching is therefore the fundamental blinding procedure in drug trials, providing the basis for simultaneous blinding of patients, treatment providers and outcome assessors.

Pharmacological interventions have different preparation formats such as tablets, capsules, aerosols, liquid, or ointment, and may contain substances that affect taste, texture, appearance, viscosity, or odour. Matching procedures may differ; for example, tablets may be embedded in identically appearing capsules, or specific flavours may be added to oral medicaments to mask a distinct taste, or similar opaque syringes may be used for injection to convey otherwise distinguishable solutions [9]. Rarely, active placebos are used to mimic the side effects of the experimental intervention [10]. More common is the double-dummy approach (i.e. active A + placebo B vs. placebo A + active B) for trials involving comparisons between two or more experimental treatments with differing routes of administration [9].

Such matching procedures are often challenging to design, may be time-consuming and increase the cost of trials. The scientific ideal of developing fully matched interventions will often have to be weighed against the concrete increase in cost and logistic challenges when planning and running a trial. It is therefore not surprising that matching procedures in drug trials are not always successful. For example, in a randomised trial of oral typhoid vaccine vs. placebo investigators discovered that taste and aroma was distinct for the experimental vaccine [11]. In another trial of oral zinc vs. calcium lactate placebo for common cold, Eby and colleagues reported that about half of the patients randomized to the experimental zinc treatment noted the telltale metallic aftertaste of zinc [12].

However, we do not know how often inadequate matching occurs in drug trials. A reliable assessment of the proportion of drug trials with inadequate matching would be of interest to readers of trial publications, to funders, to investigators running a trial and to researchers conducting systematic reviews and formally assessing the risk of bias due to inadequate blinding.

Thus, we decided to systematically review matching quality studies. Our primary objective was to assess the proportion of studies that concluded inadequate matching; our secondary objective was to describe mechanisms for inadequate matching.

## Methods

### Search strategy

**Fig. 1:**
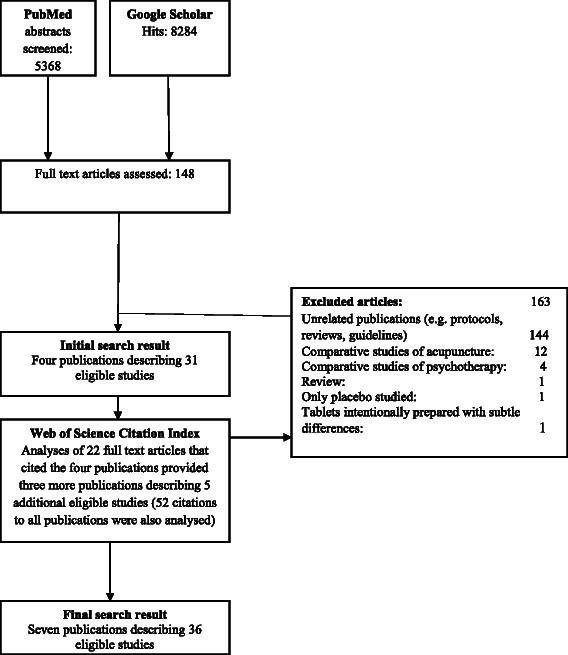
PRISMA Flow of database search for identifying eligible studies

We searched PubMed and Google Scholar from inception onwards. Our core search string was “clinical trial” AND “(blind* OR mask* OR match*) AND (drug OR placebo) AND (taste OR smell OR appearance) (Additional file 1). We searched references of eligible studies obtained and used Web of Science Citation Index to access publications that referenced the initial “seed articles” (Fig. 1). Our last formal search was conducted on 14 April, 2014.

### Eligibility criteria

We included studies of matching quality, i.e. studies that assessed whether supposedly indistinguishable experimental and control drug interventions from randomized clinical trials could be distinguished due to physical properties (e.g. colour and appearance, smell, taste, or texture). We included studies where a group of assessors was asked to identify differences in one or more interventions pairs (for example experimental and control tablets) from one or more trials, and asked to categorize the interventions (either into two different interventions or into experimental and control). We also included pilot trials of treated patients or volunteers, but only if it was very unlikely that patients or volunteers could identify intervention type through their pharmacological effect. For example, in a study of zinc for common cold, healthy participants took interventions in a pilot study of only 14 h [13].

We excluded studies of device interventions (for example acupuncture) or psychological interventions, studies involving assessments of the properties of a single trial intervention (e.g. control only) and experimental studies where the compared interventions were intentionally prepared with differences (and not intended for use in a real clinical trial) (Fig. 1).

### Data extraction

One author (SB) scanned titles and abstracts from PubMed and text fragments from Google Scholar, retrieved and assessed full texts of potentially eligible studies.

Based on a pre-tested form, two authors (SB, AH or MW) independently extracted data on the experimental and control interventions, the clinical conditions concerned, the formulation types, physical properties compared, the description of comparison methods, information on who developed or provided the compared interventions, descriptions of persons involved in the assessments, main outcomes assessed, any statistical analysis, and the authors’ conclusion.

### Analysis

We anticipated substantial methodological differences between the matching quality studies; for example the criteria for deciding inadequate matching, statistical approach, the number and types of sensory qualities which were assessed, number of assessors, the information given to assessors, and the pharmaceutical preparation formats. We therefore planned to summarize the data qualitatively without a formal meta-analysis.

We based our primary analysis on the conclusions of each study report.

As a basis for supportive analyses we defined both a low and a high threshold for inadequate matching. We defined “low threshold” for inadequate matching to mean that the interventions were considered inadequately matched if at least one assessor (of a group of assessors) had found obvious differences between the interventions, or if a sensory quality had been left unexamined in a matching study because a good match was regarded unfeasible (for example if taste was excluded from a study where the experimental intervention had a distinctly bitter taste). In case this information was not available in a study, we defined “low threshold” pragmatically on a case-to-case basis. A high threshold meant that the interventions were considered inadequately matched only if more than 75 % of assessors had found obvious differences between the interventions. In case this information was not available in a study, we defined “high threshold” pragmatically on a case-to-case basis.

## Results

We read 170 full articles based on 5368 PubMed abstracts, 8284 hits from Google Scholar, and 52 citations in Web of Science Citation Index (Fig. 1). We excluded 163 full text articles (Fig. 1). Thus, we identified seven articles [13–19], published from 1974 to 2011, describing a total of 36 eligible matching quality studies.

### General characteristics

The 36 trials involved were mainly conducted in psychiatry, cardiology, pulmonology and infectious diseases (Table 1). The most frequently compared formulation type was tablet. The majority of the compared interventions were provided by pharmaceutical companies (Table 1). In most cases the assessment of the matching quality was conducted by a small group of persons recruited from the investigation team or volunteers (e.g. students and administrative staff members) (Tables 2). Twenty-eight trials (78 %) were conducted before 1977.

**Table 1:** Characteristics of included matching quality studies

Characteristics	n = 36 (%)
Specialty	
Psychiatry	6 (17)
Cardiology	5 (14)
Respiratory medicine	5 (14)
Infectious diseases	5 (14)
Neurology	2 (6)
Rheumatology	2 (6)
Dermatology	2 (6)
Complementary-alternative medicine	2 (6)
Gastroenterology	1 (3)
Medical^a^	3 (8)
No information	3 (8)
Formulation types	
Tablet	17 (47)
Capsule	12 (33)
Liquid (injection and oral medicament)	2 (6)
Ointment	2 (6)
Aerosol	2 (6)
Nasal spray	1 (3)
Type of control intervention	
Placebo	33 (92)
Active	3 (8)
Producer of the interventions	
Pharmaceutical company	25 (69)
Investigators	1 (3)
Not reported	9 (25)
Pharmacy	1 (3)
Method of comparison	
Group of assessors	
Assessors n = 4	26 (72)
Assessors n = 32-52	8 (22)
Pilot trial	2 (6)

^a^Specific medical sub-specialties unclear

**Table 2:** Characteristics of included publications

Paper	General information	Assessment methods	Drug format (Number trials), sensory quality	Analysis	Basis for deciding inadequate matching
Hill and colleagues, 1976	22 trials (11 parallel group trial and 10 cross-over trial), described as “double-blind”, conducted in the UK over 12 months in 1973–74 and published in four journals (e.g. BMJ and Lancet). Out of 32 eligible trials 13 did not participate. Of the 19 remaining trials 3 were multi-armed, providing a total of 22 trial comparisons. In 19 trials the control was placebo. Drug companies produced the trial interventions	Four assessors (medical and administrative staff) were twice provided with 4 interventions from each trial. The assessors were “without knowledge of the experimental design” The 4 interventions consisted of 2 experimental and 2 controls), and assessors conducted 6 comparisons independently: (2 × the same interventions [e.g. control vs. control] and 4 × matched interventions [intervention vs. control]). In total, each assessor did 4 comparisons per trial of identical interventions and 8 comparisons of matched interventions. The number of sensory qualities involved differed depending on drug format, providing a potential number of assessments per trial with tablets or capsules of 64 (identical interventions) and 128 (matched interventions)	Tablet (11), colour, shape and appearance, smell, taste	For each trial, the proportion of assessments reported as different for identical interventions (control vs. control) (false positive rate) was subtracted from the proportion of assessments reported as different for the matched interventions: 35 %, 16 %, 41 %, 34 %, 6 %, 42 %, 43 %, 49 %, 15 %, 43 %, 60 %, 60 %, 42 %, 69 %, 27 %, 0 %, 0 %, 0 %, −2 %, 1 %, 20 %, 59 %	Based on a qualitative assessment the investigators decided which trials were inadequately matched (“obvious differences” were detected by all 4 assessors)
			Capsule (5), colour, shape and appearance, smell, taste		
			Aerosol and nasal spray (3), the container, smell and taste of aerosol		
			Ointment (2), colour, appearance and consistency, smell		
			Liquid (1), the ampoule, colour and liquid consistency		
Blumen-thal and colleagues, 1974	6 trials (type not reported), described as “double blind”. Trials were selected from those the investigators had been involved in because research assistants had noted differences between experimental and controls In all six trials the control was placebo. It was not reported who produced the trial interventions	Fifty-two assessors (Students, spouses, lab scientists, secretaries) served as assessors. In one part of the study (“patient-simulated”) 32 assessors examined a container with 6 trial interventions from each trial independently (from 5 trials: 3 experimental and 3 controls, and in one trial: 2 experimental of one type, 2 experimental of another type and 2 controls). Assessors were instructed to decide whether interventions were all one type or not, and to categorize them accordingly. The second part of the study (“experimenter-simulated”) involving 20 assessors was identical to the first part, but they were told specifically that the 6 interventions definitely consisted of both experimental and controls.	Tablet (4), Sensory qualities not specified.	For each trial, the proportion of assessments for which assessors correctly separated all experimental intervention from placebo intervention was calculated. For the “patient-simulated study” the proportions were: 19 %, 36 %, 82 %, 19 %, 0 %, 100 % For the “experimenter-stimulated study” the proportions were: 90 %, 85 %, 85 %, 90 %, 0 %, 100 %	Based on a Chi^2^ test the authors decided for each trial whether assessors were able to differentiate better than chance between experimental and control interventions.
			Capsule (1), Sensory qualities not specified.		
			Liquid (1), Sensory qualities not specified.		
Walter and colleagues, 2005	1 trial (parallel group), described as “double-blind”. The study was a pre-trial assessment of blinding integrity. The control was placebo. Drug company produced trial interventions	Four assessors (“study investigators”) were asked to examine independently a package of 10 tablets. The assessors were informed that it contained an equal number of experimental and control interventions.	Tablet (4), Taste and appearance	The number of correctly assigned tablets were recorded for each assessor	Based on a Chi^2^ test the authors decided whether assessors were able to differentiate better than chance between experimental and control interventions.
Fai and colleagues, 2011	1 trial (parallel group), described as “double-blind”. The study was a pre-trial assessment of blinding integrity. The control was placebo. The trial investigators produced the trial interventions.	49 “adults” served as assessors. It was not reported how many interventions each assessors examined or what information was provided to assessors. In one part of the study 15 assessors examined the packaging (box and bottle). In a second part of the study 11 assessors examined the capsules. In a third part of the study 23 assessors tested “the overall result” by guessing whether an intervention was control or experimental.	Capsule (1), The appearance and texture of package and capsule, and smell of capsule content. Taste was excluded from the assessment because “the taste was not adjusted with bitter agent”	Each assessor of packaging and capsules scored each property (exactly identical = 3 very close to unanimous = 2; significant difference = 1; inconsistent 0). The “overall result” was based on a comparison between actual intervention status and guessed status.	Based on Fischer’s exact test the authors decided whether assessors were able to differentiate better than chance between experimental and control interventions.
Dupin-Spriet and colleagues, 1993	3 trials (type not reported) The control was placebo Not reported who provided the trial interventions	Four assessors (type not reported) were provided with 8 pairs of tablets from each trial. The information, if any, provided to the assessors on the distribution of experimental and control interventions was not reported. The 8 intervention pairs consisted of 4 matching pairs (experimental vs. controls), and 4 identical pairs (e.g. control vs. control)	Tablet (3), appearance (shape, colour, surface)	For each trial, the proportion of assessments reported as different for the identical interventions (false positive rate) was subtracted from the proportion of assessments reported as different for the matched interventions:	Based on Fischer’s exact test the authors decided for each trial whether assessors were able to differentiate better than chance between experimental and control interventions.
Wen and colleagues, 2004	1 trial (parallel group) Pharmacy provided trial intervention	32 assessors (e.g. doctors, nurses, graduate students) were provided with two bottles of experimental and control interventions that they examined independently. One group of 10 assessors performed “placebo test”. One group of 22 assessors performed a “simulative test”. It was not reported whether the experimental and control interventions were paired during the assessments	Capsule (1), bottle and capsule appearance, bottle label and notes, size, shape, quality, colour, smell, taste, and capsule content	Proportion of assessments found “not uniform” on specific qualities and overall was calculated.	Based on Fisher’s exact test the authors decided whether assessors were able to differentiate better than chance between experimental and control interventions.
Farr and colleagues, 1987	2 pilot-trials (parallel group) Drug company provided intervention The control was placebo	Two pilot-trials (low and high dose zinc) randomizing assessors (students and university employees) to experimental or control. Assessors were informed that one of the compared substances had shown possible effect, and that they would be asked to decide whether they were receiving an “active” or an “inactive” compound. In the first pilot-trial 224 assessors were allocated to 30 mg zinc or placebo (0.0004 mg denatonium benzoate) In the second pilot-trial, 300 assessors were allocated to 23 mg zinc or placebo A (denatonium benzoate 0.00125 mg) or placebo B (denatonium benzoate 0.0025 mg) Assessors ingested tablets 8 times over one day (14 h). After the fifth tablet assessors filled out a structured questionnaire.	Tablet (2), bitter taste, aftertaste, palatability and guesses as to whether they believed they were receiving active or placebo.	Proportions of each assessment (e.g. bitter taste) was compared between trial groups (i.e. experimental and control groups)	Based on a Chi^2^ test the authors decided whether proportions of each assessments (experimental and control) differed more between trial groups than expected by than chance.

Twenty-two matching quality studies were based on a defined cohort of randomized clinical trials. The cohort consisted of all “double-blind” trials conducted in the UK within one year (August 1973 to August 1974), and published in The Lancet, BMJ, Clinical Allergy or Current Medical Research and Opinion (the “UK trial cohort”) [16].

Fourteen other matching quality studies were not based on a defined trial cohort.

### Primary analysis

Inadequate matching was concluded for 16 of the 36 (44 %) trials (Table 3). The mechanisms involved were typically differences in taste (17/36; 47 %), colour (15/36; 42 %), and appearance (e.g. texture, shape, consistency) (13/36; 36 %).

**Table 3:** Main and supplementary results

Paper	Main results: proportion of trials with inadequate matching based on the conclusion of the publications	Supplementary results: Proportion of trials with inadequate matching based on high/low thresholds^a^	
		High threshold	Low threshold
Hill and collegues, 1976	7 of 22 trials	6/22 trials	14/22 trials
Blumenthal and colleagues, 1974	5 of 6 trials	5/6 trials^b^	5/6 trials
Walter and colleagues, 2005	1 of 1 trial	0/1 trial	1/1 trial
Fai and colleagues, 2011	0 of 1 trial	0/1 trial	1/1 trial^c^
Dupin-Spriet and colleagues, 1993	1 of 3 trials	0/3 trials	3/3 trials
Wen and colleagues, 2004	1 of 1 trial	1/1 trials	1/1 trials
Farr and colleagues, 1987	1 of 2 trials	0/2 trials^d^	1/2 trials^e^

^a^The high thresholds are in most cases based on at least 75 % of assessors finding clear differences. The low thresholds are in most cases based on 25 % of assessors finding clear differences

^b^Two groups of assessors were involved. In one group (patient-simulated), only in one trial did 75 % or more of assessors find clear difference between experimental and control intervention. In the second group of assessors (experimenter-simulated), over 80 % of assessors found clear differences in five out of six experimental and control drugs

^c^Taste was excluded from the study because “taste was not adjusted with bitter agent” though the experimental intervention most likely had a more bitter taste

^d^Based on chance alone, 50 % of assessors would be expected to correctly guess intervention types. We defined high threshold to imply that at least 75 % of assessors randomized to the experimental intervention perceived being on the experimental intervention

^e^Significantly more than 50 % of assessors randomized to experimental group perceived to have received the experimental intervention

### Secondary analyses

Based on a high threshold for inadequate matching, 12 of the 36 trials (33 %) were inadequately matched (Table 3). Based on a low threshold for inadequate matching, 26 trials (72 %) were inadequately matched (Table 3).

Inadequate matching was concluded for 7 of 22 trials based on the UK trial cohort (32 %). In contrast, inadequate matching was concluded for 9 out of 14 trials (64 %) not based on a trial cohort.

The proportion of inadequate matching did not seem to depend on publication year. Trials published before 1977 had inadequate matching in 4 of 10 trials (12/28;43 %). This was also the case for trials published between 1977 and 2000 (2/5;40 %), and for trials published after 2000 (2/3;67 %).

### Results of the individual publications

Hill and colleagues [16] studied supposedly matching interventions from a defined cohort of 22 trials (i.e. the UK trial cohort, see above). Samples of intervention pairs included different formats, for example tablets and capsules. A group of four assessors independently compared matched interventions (i.e. experimental-control) (Table 2). In addition, randomly mixed into the sequence of such intervention pairs, the same assessors compared a number of identical interventions (e.g. control-control). The assessors were “without knowledge of the experimental design”. For each trial, the proportion of assessments reported as different for the truly identical intervention pairs (e.g. control–control) was a measure of observer error (i.e. false positive fraction), and was subtracted from the proportion of assessments reported as different for the matched interventions. No formal statistical analysis was conducted, and the decision of inadequate matching was based on a qualitative assessment. The investigators’conclusion was inadequate matching in seven of 22 (32 %) trials.

Blumenthal and colleagues [17] studied supposedly matching interventions from six trials the investigators had been involved in. The trials compared antidepressants or anxiolytics to placebos (content not reported). The drug formats were tablet (four trials), capsule (one trial), and oral liquid (one trial). There were two groups of assessors. One group of 32 assessors was asked to decide whether six interventions (e.g. tablets) from each trial differed, and if so, to categorize interventions into the two groups (Table 2). A second group of 20 assessors were specifically told that the six interventions consisted of different types. Both groups of assessors were able to differentiate between experimental and placebo interventions significantly better than chance in five trial interventions, the exception being an antidepressant capsule used in one trial. A few assessors (number not specified) also tasted the interventions and reported that “the active medications” numbed the tongue. The investigators’ conclusion was inadequate matching in five out of the six trials.

Walter and colleagues [15] studied supposedly matching oral antibiotic (amoxicillin) and placebo (type not reported) tablets for childhood pneumonia from one randomized trial [20]. Four assessors were asked to examine a package containing ten tablets and to divide them into two groups of five each thought to contain the same drug type (Table 2). The primary concern was whether the interventions tasted differently. The investigators concluded adequate matching based on taste. However, two of the four assessors were unexpectedly able to distinguish between the antibiotic and placebo based on the appearance. Thus, the investigators’ conclusion was inadequate matching.

Fai and colleagues [14] studied supposedly matching herbal preparations (Danshen and Gegeng) and placebo (starch with caramel) for the prevention of cardiovascular disease from one randomized trial [21]. Three groups of assessors conducted the study; a group of 15 assessors examined the appearance and texture of box and bottle, a second group of 11 assessors examined the appearance and texture of capsule and a third group of 23 assessors tested “the overall result” by guessing whether an intervention was experimental or placebo. Taste was excluded from the assessment because “the taste was not adjusted with bitter agent”. The herbal and placebo interventions were reported as “close to uniformity” in package and labeling; capsules were “very identical” and the overall comparison indicated that there were no detectable differences between herbal and placebo preparations in the properties assessed. The investigators’ conclusion was adequate matching based on properties tested.

Dupin-Spriet and colleagues [18] studied supposedly matching tablets (content not reported) and placebo (type not reported) for clinical conditions (not reported) from three randomized trial. There was no information on instructions given to assessors. A group of four assessors were asked to examine eight pairs of tablets consisting of four matching pairs and four identical pairs, and to note possible differences in appearance (Table 2). The assessors were able to differentiate between the experimental and placebo tablets in one of the three pairs. The investigators ‘conclusion was inadequate matching in one out of three trials.

Wen and colleagues [19] studied a supposedly matching herbal preparation (Shengmai capsule) and a placebo (type not reported) for chronic heart failure in one randomized trial. One groups of ten assessors performed a “placebo test” involving examination of the appearance, bottle label, odour, taste, capsule content. Another group of 22 assessors performed a “simulative test” involving examination of overall uniformity of experimental and placebo interventions. Each assessor examined “two bottles” of experimental and placebo capsules (Table 2). The experimental intervention was found to be identical to placebo in bottle appearance, label and capsule but to differ in odour, taste and texture of capsule contents. The investigators’ conclusion was inadequate matching.

Farr and colleagues [13] conducted two pilot-trials of zinc gluconate tablets (30 mg tablet for high-dose test I; 23 mg for low-dose test II) and supposedly matching placebos (dermatonium benzoate) for common cold. These two-pilot trials preceded the subsequent two full trials [22]. In the first pilot-trial 224 assessors were allocated to 30 mg zinc or placebo (0.0004 mg denatonium benzoate). In the second pilot-trial, 300 assessors were allocated to 23 mg zinc or placebo A (denatonium benzoate 0.00125 mg) or placebo B (denatonium benzoate 0.0025 mg). Assessors ingested tablets 8 times over one day (14 h), and filled out a structured questionnaire (Table 2). They were informed that one of the tablets (but not which one) had shown possible efficacy in the treatment of common cold and that they would be asked to decide whether they were receiving an active or inactive tablets based on the taste, aftertaste and palatability. For the high-dose test, assessors randomised to 30 mg zinc were more likely to report intervention as bitter and unpleasant and thus concluded they were on experimental intervention. For the low-dose taste test, assessors randomised to 23 mg zinc were less likely to report the intervention as bitter with no significant difference in proportion of zinc and placebo recipients believing they were on experimental intervention. The investigators’ conclusion was inadequate matching in one out of two pilot-trials.

## Discussion

We identified 36 studies of the matching quality of experimental and control interventions in blinded randomised drug trials. Sixteen of the 36 studies (44 %) concluded inadequate matching. The proportion of inadequate matching seemed to vary according to study design, especially variations in thresholds for inadequate matching and whether a study was based on a defined cohort of trials or not. Typical mechanisms of inadequate matching were differences in taste or colour. Studies of matching quality in contemporary trials were rarely published but showed similar results as for older trials.

### Strengths and weaknesses of the study

To our knowledge, this is the first systematic review of studies that investigated the quality of matching of drug interventions in blinded randomised clinical trials. We identified a fairly large number of studies, which involved a broad range of pharmacological formulations and methodological approaches.

Studies of matching quality in randomized trials are rarely published, and are not routinely reported in a way that makes them easily accessible through standard literature searches. We developed a detailed search strategy involving both a full-text database and Web of Science Citation Index. However, we cannot exclude that some studies have not been identified, especially unpublished comparisons [23].

Twenty-eight of the 36 studies we did identify were based on trials conducted before 1977. The blinding procedures in these early trials are not necessarily representative for blinding procedures in more contemporary ones. However, blinding was considered one of the most important methodological aspects of a randomized trial during the 1970′s, so it is likely that the matching procedures were well prepared in many cases. Also, we found no clear tendency for contemporary trials to more often have adequate matching, though this was based on a modest number of studies.

The methods of the included matching studies varied considerably. The proportion of inadequate matching in studies based on a defined trial cohort (i.e. the UK trial cohort) was lower (32 %) than the proportion of inadequate matching in studies not based on a trial cohort (64 %). Possible explanations for this variation are analysis methods and publication bias. In the UK trial cohort calculation of inadequate matching involved a correction for false positive assessments (see Results), implying a more conservative assessment compared to most of the other studies. Furthermore, the risk of publication bias may be substantially higher in trials that are not part of a cohort as they may tend to conduct and report comparisons more frequently when differences between the physical properties of treatments were suspected and/or observed. For example, Blumenthal and colleagues initiated their study of six trials after “research assistants noticed that the active drugs could be distinguished from the placebos by small differences in physical properties” [17].

The thresholds for inadequate matching differed from study to study. For example, the threshold for inadequate matching implemented by Blumenthal and colleagues [17] involved the correct separation of all experimental from control interventions by each assessor. Other studies implemented less strict criteria. For example Walter and colleagues, considered statistically significant assignment of experimental and placebo tablets into respective groups by assessors and not necessarily correct assignment of all the tablets [15]. In a supportive analysis, we applied a high threshold for defining inadequate matching, and still found that about 1 out of 3 trials had not adequately matched interventions. Thus, the exact proportions will differ depending on which criteria for inadequate matching is adopted, but our overall qualitative conclusion is robust to threshold variations.

All the studies except the UK cohort employed significance tests to decide whether there was sufficient evidence of poor matching (Table 2). While laudable in principle, it means that the individual experimental setup (number of observations and other design details) affects conclusions, making generalizations challenging. In particular, studies with few assessors and assessments have a high risk of overlooking an existing mismatch (i.e. type 2 error). Nonetheless, many of our trials do reach a conclusion of inadequate matching despite being fairly small. The authors of the UK cohort, on the other hand, try to judge by the presumed medical importance of the detection rates, but their subtraction of a false positive fraction (Table 2) may lead to underestimation (Additional file 1). More methodological work is needed to establish the best approach.

### Other studies

The problem of inadequate matching has primarily been discussed in the context of non-pharmacological trials. Studies of matching in that class of trials have reported findings comparable to ours [24–27]. For example, in some studies comparing the perceptible physical properties of real vs. sham acupuncture (e.g. perception of needle skin penetration) have reported that participants were able to differentiate between experimental and control treatments [24, 25], while other studies have found that participants could not differentiate between the two types of interventions [26, 27].

In a previous study we analyzed 300 randomised clinical trial publications and found none that reported a pre-trial matching quality study [28]. In our present study we also find that few matching quality studies are published compared to the number of randomized clinical trials adopting matching. We suspect, however, that matching studies are carried out much more often as a quality assurance exercise before starting a randomized trial, but that the vast majority of these studies remain unpublished.

### Mechanism

Good manufacturing practice guidelines recommend strategies aimed at reducing the risk of mix-ups between experimental and control interventions [29]. This suggests that drug companies (and other producers of drug trial interventions, such as pharmacies) sometimes fail to standardize their manufacturing process. Experimental and control interventions may be produced at different sites, or not in sequence. For example, placebo production may be postponed until trial contracts are signed or is relegated to a different plant or to subcontractors. Subtle but noticeable differences between the experimental and the control interventions could easily be the result.

It is striking that trial investigators are sometimes unaware of the physical aspects of trial intervention properties that may be inadequately matched. For example, Walter and colleagues were mainly concerned about the possibility of patients identifying the experimental intervention through taste [15]. However, unexpectedly, two of four assessors were able to correctly differentiate between trial interventions based on appearance. In real clinical trial settings, other potential sources of unblinding may derive from side effects of treatments and suspicion arising from awareness of laboratory results.

It is important to point out that inadequate matching does not automatically mean loss of blinding. Loss of blinding, i.e. unblinding, involves the correct identification of which type of trial intervention is the experimental and which is the control, and not only the ability to identify a difference between two types of interventions. Only two of the 36 studies involved the identification of which intervention was experimental and which was control [13, 14], though some assessors in a third study did so without being asked to do it [17].

Pre-trial assessments provide an opportunity for correcting inadequate matching [13–15]. We included three pre-trial assessment studies. One of the three studies produced evidence of inadequate matching [15], but the members of the data monitoring committee and the principal investigator decided to proceed with the trial without correcting the flaw. The trial was published in the BMJ in 2004 [20], but the trial publication did not report assessment of matching quality, nor was inadequate matching mentioned by the authors when they summarized strengths and weaknesses of the trial [20]. In another study, taste was excluded from the assessment though there was a substantial risk that the experimental intervention had a distinctly bitter taste. This was not reported in the publication describing the trial [21]. The last pre-trial assessment study found inadequate matching in one out of two pilot trials. In the subsequent full trials [22], the authors therefore used the adequately matched formulations.

An unanswered question is which degree of bias is induced by inadequate matching. Clearly, the degree of bias in trials with partially unsuccessful matching is expected to be lower than in trials with no blinding at all. Still, it is unclear how much lower. For a significant bias to occur due to unblinded patients, a substantial proportion of patients have to note that interventions are inadequately matched and correctly deduce which is the experimental intervention. This probably does occur regularly, especially in trials with clearly inadequate matching and where both types of interventions are presented to patients, for example cross-over trials and split-body designed trials. Parallel group trials with run-in periods (placebo or experimental) or with double-dummy procedures may also be at risk. However, without a direct comparison inadequate matching is more difficult to note for a patient. A standard parallel group trial without run-in periods or double-dummy procedures may therefore be fairly robust to patient unblinding due to imperfect matching.

In contrast, the risk of bias caused by unblinding of health care personnel and outcome assessors due to inadequate matching may be substantial also in standard parallel group trials. Investigators normally have easy and repeated access to both types of interventions, so minor imperfections in matching will more readily be noted, and the unblinding of just one or a few persons may easily affect a large number of patient assessments. It is noteworthy that Bluhmenthal and colleagues found a more pronounced degree of inadequate matching in their “experimenter-simulated” substudy where assessors were specifically told that the interventions they were assessing consisted of both experimental and control interventions than in their “patient-simulated” substudy where assessors did not know whether the interventions were all of the same type [17].

The apparently first two published studies of the matching quality of pharmacological experimental and control interventions were prominently published in the 1970s [16, 17]. Surprisingly, their disturbing early results were never followed up comprehensively by subsequent studies of contemporary trial cohorts. We have only identified three studies published since 2000 (with findings consistent with that of the earlier studies). This lack of interest is surprising when considering the traditional important role of blinding in trial methodology. Nor is it in accordance with Guidelines on Good Manufacturing Practice of Investigational New Drugs for Clinical Trials, which emphasize verification of the effectiveness of blinding by a check on the physical similarity of experimental and control preparations [30–32]. Regardless, it appears that since the late 1970′ies the subject of matching in drug trials has largely been withdrawn from the scientific literature and the accompanying academic scrutiny. Today the responsibility for designing and manufacturing matched pharmacological preparations for clinical trials is dominated by the pharmaceutical industry. Furthermore, interpretations of any assessments of the adequacy of matching in clinical drug trials will often be an internal trial procedure. The results of such assessments, and any action resulting from the assessment, are rarely communicated to others.

### Implications

Our study provides an empirically based framework for the interpretation of results from blinded randomized clinical trials of drug interventions.

We interpret the exact proportions of inadequate matching with reservation. The studies based on a defined trial cohort were not contemporary, and the newer studies may involve a considerable risk of publication bias. Still, the findings are clearly disconcerting. If our result can be reproduced in a study of contemporary and representative trials, an important methodological limitation in current trial methods needs to be corrected.

We furthermore suggest that trial investigators closely examine the matching properties of the compared treatments. Such pre-trial comparisons of matched preparations could reveal correctable flaws in the matching thus reducing the risk of unblinding. We also suggest that when such examinations are carried out, the methods and results are published, for example in an Additional file 1 to the trial.

It is not entirely clear how matching studies are best conducted and analysed. Further methodological research is therefore warranted to assist researchers conducting and analyzing results from randomized clinical trials that compare matching interventions.

## Conclusion

In this systematic review we identified studies of the quality of matching of drug interventions in 36 randomized clinical trials. Sixteen of the 36 studies (44 %) concluded inadequate matching. Typical mechanisms of inadequate matching were differences in taste or colour. Studies of matching quality in contemporary trials are rarely published, but show similar results as found for older trials. Inadequate matching in drug trials may be more prevalent than commonly believed.

## Additional file

Additional file 1
**Search Strategy and Statistical Considerations** (DOCX 14 kb)
