# Supplementary material for: The matching quality of experimental and control interventions in blinded pharmacological randomised clinical trials: a methodological systematic review
Source: BMC Med Res Methodol. 2016 Feb 13;16:18. doi: 10.1186/s12874-016-0111-9 (PMC4752749; doi:10.1186/s12874-016-0111-9)
Supplement: Additional file 1 — Search Strategy and Statistical Considerations (DOCX 14 kb) [file 12874_2016_111_MOESM1_ESM.docx]

**Additional file 1**

**Search strategy**

*PubMed*

1. Clinical trial
2. Blind* OR mask* OR match*
3. Drug OR placebo OR medication OR preparation OR pair OR tablet OR ingredient OR agent OR intervention OR sham OR fake OR dummy OR mock OR vehicle OR treatment
4. Compar* OR contrast* OR inspect* OR examin* OR observ* OR diffrentiat* OR distinguish* OR difference OR evaluat* OR assess*
5. Colour OR taste OR smell OR appearance OR consistency OR shape OR container OR packag* OR fragrance* OR flavour OR flavor OR credibility OR integrity OR odour OR thickness OR texture
6. panel OR observer OR volunteer OR patient OR investigator
7. #1 AND #2 AND #3 AND #4 AND #5 AND #6

*Google Scholar*

1. Clinical trial AND blinded AND drug AND placebo AND taste AND smell AND appearance AND panel
2. Clinical trial AND match AND drug AND placebo AND taste AND smell AND appearance AND panel
3. Clinical trial AND mask AND drug AND placebo AND taste AND smell AND appearance AND panel
4. Clinical trial AND blinded AND colour AND texture AND odour AND differentiate AND panel
5. Clinical trial AND match AND colour AND texture AND odour AND differentiate AND panel
6. Clinical trial AND mask AND colour AND texture AND odour AND differentiate AND panel
7. Clinical trial AND blinded AND drug AND placebo AND physical properties AND distinguish AND (compare OR contrast OR inspect OR examine OR observe OR differentiate OR distinguish OR difference OR evaluate OR assess)
8. Clinical trial AND match AND drug AND placebo AND physical properties AND distinguish AND (compare OR contrast OR inspect OR examine OR observe OR differentiate OR distinguish OR difference OR evaluate OR assess)
9. Clinical trial AND mask AND drug AND placebo AND physical properties AND distinguish AND (compare OR contrast OR inspect OR examine OR observe OR differentiate OR distinguish OR difference OR evaluate OR assess)

**Statistical considerations**

The strategy of subtracting an empirical false positive fraction from the empirical fraction of inadequately matched intervention pairs, as done by Hill and colleagues (1), may lead to an underestimation of the actual detectability (i.e. sensitivity) of inadequate matching, as shown below.

Assume that the assessor tests for *any* kind of difference. Consider furthermore a dataset in which the truly identical intervention pairs are assessed to be different in some proportion (for example 64% of the times) and the truly matched intervention pairs are judged to be different in a lower proportion (for example 21% of the times).

Thus, when given two *truly matched interventions*, the assessor will conclude *some* kind of difference once in a while. In our example this happens 21% of the time. Furthermore, the assessor may give reasons for the verdict, and, for example, explain that for 1% of cases the perceived inadequate matching was due to an alleged colour differences and for the remaining 20% of cases the perceived inadequate matching was due to other factors (i.e. not related to colour differences).

When given two *truly non-matched interventions*, the assessor will spot a difference some of the times but not always. The typical situation will be that the inadequate matching is due to a subtle imperfection (for example a small colour difference), which the assessor would detect with a certain threshold, or sensitivity. The sensitivity could, for example, be 0.55, i.e. out of 100 truly different interventions the assessor will spot the difference in 55 cases, on average.

Note that the assessor will still report an additional spurious non-colour mismatch with probability 0.20. Now, assume for simplicity that the two aspects are independent. In this scenario, the probability that the assessor concludes adequate matching (i.e. observes *no* difference, *neither* a non-colour *nor* a colour difference) is (1 – 0.20) (1 – 0.55) = 0.36. So, with a probability of 0.36 the assessment of the intervention pairs will be ‘adequate matching’. It also follows that with a probability of 1 – 0.36 = 0.64 the assessment will be ‘inadequate matching”.

If this is the mechanism that lies behind the observed frequencies of 64% vs. 21%, then one must conclude that the subtraction procedure: 0.64 – 0.21 = 0.43, underestimates the sensitivity of 0.55.

In our example, the underestimation can be estimated as a factor close to 0.43/0.55 = 0.82.

The core problem is that the subtraction policy overlooked the fraction (0.2)(0.55) = 11% with a combined mismatch claim (perceived *both* non-colour *and* colour difference behind a claim of ‘inadequate matching’).

A probably better approach is to have the assessors record specifically what differences they feel they have observed. The resulting cross-tabulations one may make use to home in on relevant detection rates for whatever unintentional mismatch features are present, while recognising that these are situation-specific. Alternatively, one may content oneself with noting that unlike pairs do reach perceptively higher mismatch rates than like pairs, without attaching a definite meaning to the difference.
